# Improving Detection Efficiency of SARS-CoV-2 Nucleic Acid Testing

**DOI:** 10.3389/fcimb.2020.558472

**Published:** 2020-12-22

**Authors:** Jie Zhang, Kecheng Li, Ling Zheng, Jianbo Zhang, Zhilin Ren, Tiange Song, Hua Yu, Zhenglin Yang, Li Wang, Li Jiang

**Affiliations:** ^1^ Department of Laboratory Medicine, Sichuan Provincial People’s Hospital, University of Electronic Science and Technology of China, Chengdu, China; ^2^ Department of Medical Administration, Sichuan Academy of Medical Sciences & Sichuan Provincial People’s Hospital, Chengdu, China; ^3^ Sichuan Provincial Key Laboratory for Human Disease Gene Study, Sichuan Provincial People’s Hospital, University of Electronic Science and Technology of China, Chengdu, China; ^4^ Department of Nephrology, Sichuan Provincial People’s Hospital, University of Electronic Science and Technology of China, Chengdu, China

**Keywords:** SARS-CoV-2, COVID-19, nucleic acid testing, RT-PCR, detection rate, positive rate

## Abstract

**Background:**

SARS-CoV-2 nucleic acid testing (NAT) has been routinely used for COVID-19 diagnosis during this pandemic; however, there have been concerns about its high false negative rate. We dissected its detection efficiency with a large COVID-19 cohort study.

**Methods:**

We analyzed SARS-CoV-2 NAT positive rates of 4,275 specimens from 532 COVID-19 patients in Sichuan Province with different disease severities, statuses, and stages, as well as different types and numbers of specimens.

**Results:**

The total positive rate of the 4,275 specimens was 37.5%. Among seven specimen types, BALF generated a 77.8% positive rate, followed by URT specimens (38.5%), sputum (39.8%), and feces/rectal swabs (34.1%). Specimens from critical cases generated a 43.4% positive rate, which was significantly higher than that of other severities. With specimens from patients at stable status, the SARS-CoV-2 positive rate was 40.6%, which was significantly higher than that of improved status (17.1%), but lower than that of aggravated status (61.5%). Notably, the positive rate of specimens from COVID-19 patients varied significantly from 85 to 95% during 3 days before and after symptom onset, to 20% at around 18 days after symptom onset. In addition, the detection rate increased from 72.1% after testing one throat swab, to 93.2% after testing three consecutive respiratory specimens from each patient.

**Conclusions:**

SARS-CoV-2 NAT detection rates vary with patient disease severity and status, specimen type, number of specimens, and especially disease progression. Sampling as close to symptom onset as possible, and consecutively collecting more than one respiratory specimen could effectively improve SARS-CoV-2 NAT detection efficiency.

## Highlights

As SARS-CoV-2 NAT detection rates vary from 85 to 95% within 3 days before and after symptom onset, to 20% at 18 days after symptom onset, sampling as close to symptom onset as possible could effectively improve its detection efficiency.

## Introduction

In late December 2019, an outbreak of coronavirus disease (COVID-19) caused by a novel coronavirus SARS-CoV-2, emerged in Wuhan, China and spread rapidly across China and further around the world (Chen et al., 2020; Huang et al., 2020; Zhu et al., 2020). As of May 1, 2020, there have been more than 3.18 million confirmed cases of COVID-19 with over 224 thousand deaths globally (Coronavirus disease (COVID-19) Situation Report, 2020). Accurate and rapid diagnosis of COVID-19 is crucial for disease treatment and transfection control. As the initial clinical manifestations of COVID-19 are non-specific, it is difficult to distinguish SARS-CoV-2 infection from other respiratory infections based only on clinical symptoms and signs or computed tomography (CT) imaging (Chen et al., 2020; Guan et al., 2020; Huang et al., 2020). SARS-CoV-2 nucleic acid testing (NAT), specifically real-time Reverse Transcription-Polymerase Chain Reaction (RT-qPCR) assay, as a confirmation test for COVID-19 diagnosis, has played a pivotal role in disease diagnosis, monitoring, surveillance, infection control, and prevention during the pandemic (Chu et al., 2020; Corman et al., 2020; Dennis Lo and Chiu, 2020; Guan et al., 2020; Lu et al., 2020).

However, the detection efficiency of SARS-CoV-2 NAT has been criticized due to a high false negative rate (60–70%) (Rainer et al., 2004; Fang et al., 2020; Wang P. et al., 2020; Wang Y. et al., 2020). Recent studies demonstrated that the positive rates of RT-qPCR tests for SARS-CoV-2 vary significantly between specimen types (Pan et al., 2020; Wang W. et al., 2020; Xie et al., 2020). Even among the most frequently collected specimen type, throat swabs, they only generated a 32% positive rate (Wang W. et al., 2020). In addition, four groups reported that the SARS-CoV-2 viral load in respiratory tract specimens peaked at 3, 5–6 days and 10 days soon after symptom onset in several COVID-19 cases, respectively (Peiris et al., 2003; Hung et al., 2004; Al-Tawfiq and Memish, 2020; Zou et al., 2020). Recently published data also showed that false negatives from one-time sampling were as high as 30–50% in real COVID-19 cases (Alfaraj et al., 2019). While, a large cohort study of MERS-CoV reported increasing number of specimens consecutively tested could effectively improve the positive rate of NAT (Hung et al., 2004).

Thus, to investigate whether sampling in different anatomic sites, at different disease progression stages, and with different number of samples from each patient, as well as whether patient disease severity and status contributes to the false-negative results of SARS-CoV-2 NAT, we performed a retrospective analysis of SARS-CoV-2 positive rates, based on a large cohort of 4,363 specimens from 532 laboratory-confirmed COVID-19 cases at 79 reference hospitals in Sichuan Province from January 10^th^, 2019 to March 1^st^, 2020.

## Materials And Methods

### Cases and Specimens

A large cohort of 532 laboratory-confirmed COVID-19 patients presented in Sichuan Province, China, between January 10^th^, 2020 and March 1^st^, 2020 was included in the study. All patients in our study were diagnosed with COVID-19 based on the National Clinical Guidelines of COVID-19 (Edition 7) (The National Clinical Guidelines of COVID-19, 2020) and recorded in the Sichuan Provincial Health Commission Database. The study was approved by the Sichuan Provincial Health Commission and the ethics commissions of Sichuan Provincial People’s Hospital and determined to be exempt from oversight given the use of pre-existing, de-identified data. As such, individual-level informed consent was not obtained.

All general information and clinical characteristics of the patients, as well as specimen types, sampling dates, and NAT results of 4,363 specimens were submitted to Sichuan Provincial Health Commission Database by 79 COVID-19 reference hospitals in in Sichuan Province. We extracted and reviewed the data to exclude cases and/or specimens with missing core data.

### SARS-CoV-2 Nucleic Acid Testing 

SARS-CoV-2 NAT specimens included upper respiratory tract (URT, including nasal swabs, pharynx swabs and nasopharyngeal swabs) specimens, lower respiratory tract (LRT, including sputum and BALF) specimens, feces, rectal swabs, and blood. Total RNA was extracted from the specimens with CFDA-certified manual or automatic RNA extraction methods following corresponding manufacturer instructions.

The real-time RT-PCR assays were conducted in qualified CDC and hospital clinical laboratories using CFDA-certified test kits, including DAAN gene Co., Ltd. (Guangzhou. China), Sansure biotech Co., Ltd. (Changsha, China), Shanghai zjbio-tech Co., Ltd. (Shanghai, China), BGI, Co., Ltd. (Shenzhen, China), and Geneodex, Co., Ltd. (Shanghai, China), which were accordant with WHO recommended assays targeting one, two, or three SARS-CoV-2 viral genes (RNA-dependent RNA polymerase gene, nucleocapsid protein gene and envelope protein gene) (Xu et al., 2020).

### Statistical Analysis

Descriptive statistics were reported as mean ± standard deviation or median (interquartile range) for continuous variables and as frequency (percentage) for categorical variables. All COVID-19 cases were grouped into mild, moderate, severe, and critical based on symptom severity and into stable, improved, and aggravated based on disease status in the National Clinical Guidelines of COVID-19 (Edition 7) (Alfaraj et al., 2019). All specimens were grouped into nasal swabs, pharynx swabs, nasopharyngeal swabs, sputum, BALF, feces/rectal swabs, blood, and unknown specimen groups based on specimen type, while into <0, 0–7, 8-14, 15–21, 22–28 and > 28 days after onset (d.a.o) of symptoms based on the difference in days between symptom onset and specimen collection. Missing specimen types were not excluded, but classified into other specimen type group, while missing the viral NAT results were excluded from analyses.

To compare the SARS-CoV-2 NAT positive rates of specimens between different specimen types, symptom severities, disease statuses and progression stages, we performed mixed-effects logistic regression models respectively, used an unstructured covariance structure to account for the correlations within laboratory and patient levels, and reported the odds ratios, 95% confidence intervals and *P* values. All models were adjusted for age and gender.

We considered p <0.05 as statistically significant for all analyses. All statistical tests were two-sided and analyzed using STATA version MP 15 (StataCorp LLC, College Station, TX, USA).

## Results

### Patient and Specimen Profiles

A total of 532 laboratory-confirmed COVID-19 patients from 79 reference hospitals in Sichuan Province during January 10^th^, 2019 and March 1^st^, 2020 were included in our study. As of March 1^st^, 2020, 374 (70.3%) patients were discharged, three (0.6%) were dead, and 155 (29.1%) patients remain hospitalized. There were 52.6% male cases, and there were no differences in sex ratios among the four severity groups. Of the 532 patients, the majority were moderate cases (400, 75.2%), while 49 (9.2%), 51 (9.6%), and 32 (6.1%) were the mild, severe, and critical cases, respectively (**Table 1**). The median age was 45 years with a range of 0.1 to 87 years, and the ratio of the patients above 45 years increased from 26.5 to 49.7, 62.7, and 78.1% with increasing severity (**Table 1**).

**Table 1:** Patient profile.

Characteristics	Severity, No. (%)				
	Total	Mild	Moderate	Severe	Critical
**No. of Patients**	**532**	**49 (9.2)**	**400 (75.2)**	**51 (9.6)**	**32 (6.1)**
**Male**	**280 (52.6)**	**26 (53.1)**	**203 (50.8)**	**30 (58.8)**	**21 (65.6)**
**Age, median (range), year**	**45 (0.1, 87)**	**31 (0.1, 71)**	**44 (0.25, 79)**	**48 (20, 84)**	**60 (32, 87)**
<44 year, No. (%)	263 (49.4)	36 (73.5)	201 (50.3)	19 (37.3)	7 (21.9)
≥45 year, No. (%)	269 (50.6)	13 (26.5)	199 (49.7)	32 (62.7)	25 (78.1)

A total of 4,363 specimens from the 532 patients were collected between 17 days before and 50 days after onset (d.a.o). Of the 4,363 specimens tested for SARS-CoV-2, 88 had recorded undetermined results, and were therefore excluded. Hence 4,275 specimens with SARS-CoV-2 results were eventually included in the study. Among them, the predominant specimens were 1,992 (46.6%) throat swabs, 1,009 (23.6%) sputum, and 574 (13.4%) feces/anal swabs. In addition, there were 319 (7.5%) nasopharyngeal swabs, 256 (6.0%) nasal swabs, 80 (1.9%) blood, nine (0.2%) BALF, and 36 (0.8%) others with unknown types (**Table 2**). The median number of specimens collected from each patient was six (ranging from one to 50) (**Table 2**).

**Table 2:** Specimen profile.

Specimen type	Time course of disease, d.a.o, No. (%)						
	Total	<0	0-7	8-14	15-21	22-28	>28
*^a^* **URT specimen**	**2567 (60.0)**	**90 (3.5)**	**533 (20.8)**	**710 (27.7)**	**661 (25.8)**	**376 (14.7)**	**197 (7.7)**
Throat swabs	1992 (46.6)	75 (3.8)	441 (22.1)	573 (28.8)	512 (25.7)	254 (12.8)	137 (6.9)
Nasal swabs	256 (6.0)	5 (2.0)	33 (12.9)	55 (21.5)	63 (24.6)	64 (25.0)	36 (14.1)
*^b^*NP swabs	319 (7.5)	10 (3.1)	59 (18.5)	82 (25.7)	86 (27.0)	58 (18.2)	24 (7.5)
*^c^* **LRT specimen**	1018 (23.8)	11 (1.1)	108 (10.6)	222 (21.8)	279 (27.4)	2234 (22.0)	174 (17.1)
Sputum	**1009 (23.6)**	**11 (1.1)**	**105 (10.4)**	**220 (21.8)**	**276 (27.4)**	**223 (22.1)**	**174 (17.2)**
BLAF	9 (0.2)	0 (0.0)	3 (33.3)	2 (22.2)	3 (33.3)	1 (11.1)	0 (0.0)
Feces/rectal swabs	**574 (13.4)**	**1 (0.2)**	**23 (4.0)**	**111 (19.3)**	**178 (31.0)**	**151 (26.3)**	**110 (19.2)**
Blood	80 (1.9)	0 (0.0)	8 (10.0)	20 (25.0)	28 (35.0)	13 (16.3)	11 (13.8)
Others	36 (0.8)	0 (0.0)	1 (2.8)	2 (5.6)	16 (44.4)	11 (30.6)	6 (16.7)
**Total**	**4275**	**102 (2.4)**	**673 (15.7)**	**1065 (24.9)**	**1162 (27.2)**	**775 (18.1)**	**498 (11.6)**
No. of Specimens from each patient, median (range)	**6 (1, 50)**	**1 (1, 7)**	**1 (1, 8)**	**2 (1, 16)**	**2 (1, 16)**	**3 (1, 18)**	**4 (1, 27)**

a URT, Upper Respiratory Tract.

b NP, Nasopharyngeal.

c LRT, Lower Respiratory Tract.

### Positive Rates of SARS-CoV-2 NAT in Different Specimen Types 

Out of the 4,275 specimens, 1,605 (37.5%) were detected positive for SARS-CoV-2. Among seven specimen types, BALF (77.8%, 7/9), nasopharyngeal swabs (40.4%, 129/319), and sputum (39.8%, 402/1009) had the three highest positive rates. Throat swabs (38.5%, 767/1992), nasal swabs (35.9%, 92/256), and feces/rectal swabs (34.0%, 195/574) ranked fourth, fifth, and sixth, while blood only had a 5.0% (4/80) positive rate (**Table 3**). Notably, Compared to throat swabs, BALF [OR = 6.24, 95%CI(1.08, 35.92), *P* = 0.04] and sputum [OR = 1.24, 95%CI(1.01, 1.52), *P* = 0.04)] possessed significantly higher positive rates; in contrast, feces [OR = 0.74, 95%CI(0.58, 0.95), *P* = 0.016] and blood [OR = 0.06, 95%CI(0.02, 0.16), *P* < 0.001] showed significantly lower positive rates (**Table 3**).

**Table 3:** SARS- CoV- 2 NAT positive rates in different specimens (*n* = 4,275).

Specimen type	Positive rate %(n/N)		Overall			URT vs. sputum vs. feces/rectal swabs	
			OR (95% CI)	*P* value		OR (95% CI)	*P* value
*^a^*URT specimens	**38.5 (989/2567)**					Reference	
Throat swabs	**38.5 (767/1992)**	Reference					
Nasal swabs	35.9 (92/256)	0.80 (0.57–1.12)			0.187		
*^b^*NP swabs	40.4 (129/319)	1.18 (0.86–1.60)			0.301		
*^c^* **LRT specimens**	40.2 (409/1018)						
Sputum	**39.8 (402/1009)**	1.24 (1.01–1.52)			**0.040** ^d^	1.24 (1.02–1.50)	**0.027** *^d^*
BALF	77.8 (7/9)	6.24 (1.08–35.92)			**0.040** ^d^		
Feces/rectal swabs	**34.0 (195/574)**	0.74 (0.58-0.95)			**0.016** ^d^	0.74 (0.59–0.93)	**0.011** ^d^
Blood	5.0 (4/80)	0.06 (0.02–0.16)			**<0.001** ^d^		
Others	22.2 (8/36)	0.54 (0.21–1.37)			0.194		
**Total**	**37.5 (1605/4275)**						

a URT, Upper Respiratory Tract.

b NP, Nasopharyngeal.

c LRT, Lower Respiratory Tract.

d P< 0.05 considered as significant.

There was no significant difference found in the positive rates among three URT specimens, including throat, nasal, and nasopharyngeal (NP) swabs. We then compared SARS-CoV-2 positive rates of URT specimens, sputum, and feces/rectal swabs, which were the most commonly accepted specimens for SARS-CoV-2 NAT, representing three different anatomic sites. The positive rate of URT specimens was significantly lower than that of sputum [OR = 1.24, 95%CI (1.02, 1.50), *P* = 0.025], but significantly higher than that of feces/rectal swabs [OR = 0.74, 95%CI (0.59, 0.93), *P* = 0.011] (**Table 3**).

### Positive Rates of SARS-CoV-2 NAT Under Different Disease Severities and at Different Disease Statuses 

To find whether the SARS-CoV-2 NAT positive rates vary with disease severity and status, we analyzed the positive rates of the 4,275 specimens among four disease severities and among three disease statuses. There were 34.1% (95/279), 37.3% (1237/3315), 38.1% (162/425), and 43.4% (111/256) positive SARS-CoV-2 NAT results in the mild, moderate, severe, and critical cases, respectively. Compared to the positive rate in the mild cases, only that in the critical cases was significantly increased [OR = 2.02, 95%CI (1.19, 3.41), *P* = 0.009] (**Table 4**).

**Table 4:** SARS-CoV-2 NAT positive rates under different disease severities, statuses and stages (*n* = 4275).

Patient classification	Positive rate % (n/N)	OR (95% CI)	*P* value
**Disease severity**			
Mild	34.1 (95/279)	Reference	
Moderate	37.3 (1237/3315)	1.22 (0.85–1.74)	0.279
Severe	38.1 (162/425)	1.32 (0.83–2.09)	0.241
Critical	43.4 (111/256)	2.02 (1.19–3.41)	**0.009** *^b^*
**Disease status**			
Stable	40.6 (1481/3650)	Reference	
Improved	17.1 (100/586)	0.23 (0.17–0.32)	**<0.001** *^b^*
Aggravated	61.5 (24/39)	2.81 (1.28–6.16)	**0.001** *^b^*
**Disease stage**			
<0*^a^* d.a.o	76.5 (78/102)	3.24 (1.78–5.92)	**<0.001** *^b^*
0–7*^a^* d.a.o	70.1 (472/673)	Reference	
8–14*^a^* d.a.o	33.8 (360/1065)	0.09 (0.06–0.12)	**<0.001** *^b^*
15–21*^a^* d.a.o	24.7 (287/1162)	0.03 (0.02–0.04)	**<0.001** *^b^*
22–28*^a^* d.a.o	31.4 (243/775)	0.03 (0.02–0.25)	**<0.001** *^b^*
>28*^a^* d.a.o	33.1 (165/498)	0.02 (0.01–0.04)	**<0.001** *^b^*

a d.a.o days after onset.

b P < 0.05 considered as significant.

Among three disease statuses, the SARS-CoV-2 NAT positive rates were 17.1% (100/586) under the improved status, 40.6% (1481/3650) under the stable status, and 61.5% (24/39) under the aggravated status. Compared to the stable status, the improved status had significantly lower SARS-CoV-2 positive rate with an adjusted odds ratio of 0.23 [95%CI (0.17–0.32), *P*<0.001], in contrast, the aggravated status had significantly higher positive rates with an adjusted odds ratio of 2.81 [95%CI (1.28–6.16), *P* = 0.001] (**Table 4**).

To reveal optimal specimen types in different severities and disease statuses, we analyzed the positive rates of URT specimens, sputum and feces/rectal swabs among four severities and three statuses. In the moderate cases, sputum [OR = 1.25, 95%CI (1.02–1.55), *P* = 0.036] showed a significantly higher positive rate, while feces/rectal swabs showed significantly lower positive rates in both the moderate [OR = 0.71, 95%CI (0.55–0.93), *P* = 0.012] and the critical [OR = 0.16, 95%CI (0.05–0.49), *P* = 0.001] cases (**Table 5**). At aggravated status, there was no significant difference in the positive rates among three specimen types (**Table 5**). Compared to URT specimens, sputum [OR=2.26, 95%CI (1.14–4.47), *P* = 0.019] had a significantly higher positive rate at improved status, and feces/rectal swabs [OR=0.71, 95%CI (0.55–0.91), *P* = 0.007] had a significant lower positive rate at stable status (**Table 5**).

**Table 5:** SARS-CoV-2 NAT positive rates of different specimens under different disease severities, statuses and stages.

Patient classification	*^a^*URTspecimen	Sputum			Feces/rectal swabs		
	Positive rate, %(n/N)	Positive rate, %(n/N)	OR (95%CI)	*P* value	Positive rate, %(n/N)	OR (95%CI)	*P* value
**Disease stage**	Reference						
Mild	32.6 (73/224)	21.7 (5/23)	0.54 (0.18–1.62)	0.270	63.0 (17/27)	2.52 (0.96–6.60)	0.059
Moderate	38.2 (759/1989)	39.6 (316/798)	1.25 (1.02–1.55)	**0.036** *^d^*	34.2 (153/448)	0.71 (0.55–0.93)	**0.012** *^d^*
Severe	40.8 (89/218)	41.4 (46/111)	1.40 (0.76–2.57)	0.275	31.8% (21/66)	0.92 (0.44–1.93)	0.821
Critical	49.3 (67/136)	45.5 (35/77)	0.77 (0.38–1.56)	0.463	15.2 (5/33)	0.16 (0.05–0.49)	**0.001** *^d^*
**Progressive status**	Reference						
Improved	12.0 (36/300)	23.8 (39/164)	2.26 (1.14–4.47)	**0.019** *^d^*	22.0 (20/91)	1.66 (0.75–3.67)	0.214
Stable	41.8 (937/2243)	42.7 (356/834)	1.15 (0.94–1.41)	0.184	36.5 (175/480)	0.71 (0.55–0.91)	**0.007** *^d^*
Aggravated	62.5 (15/24)	63.6 (7/11)	N/A*^b^*	0.287	33.3 (1/3)	N/A*^b^*	0.958
**Disease stage**	Reference						
<0*^c^* d.a.o	75.6 (68/90)	81.8 (9/11)	17.10 (0.76-193.34)	0.99	100.0 (1/1)	N/A*^b^*	N/A*^b^*
0–7*^c^* d.a.o	69.0 (368/533)	82.9 (87/105)	2.88 (1.18–7.02)	**0.020** *^d^*	52.2 (12/23)	0.49 (0.10–2.32)	0.370
8–14*^c^* d.a.o	31.7 (225/710)	41.4 (91/220)	1.55 (0.95–2.51)	0.076	36.9 (41/111)	0.85 (0.46–1.58)	0.614
15–21*^c^* d.a.o	23.1 (153/661)	23.9 (66/276)	1.35 (0.86–2.12)	0.195	33.7 (60/178)	1.26 (0.77–2.07)	0.353
22–28*^c^* d.a.o	27.9 (105/376)	37.7 (84/223)	2.29 (1.34–3.92)	**0.003** *^d^*	34.4 (52/151)	1.13 (0.63–2.03)	0.675
>28*^c^*d.a.o	35.0 (69/197)	37.4 (65/174)	1.11 (0.63–1.96)	0.721	27.3 (30/110)	0.42 (0.21–0.84)	**0.013** *^d^*

a ^a^ URT, Upper Respiratory Tract.

b N/A, Not available.

c d.a.o days after onset.

d P < 0.05 considered as significant.

### Positive Rates of SARS-CoV-2 NAT During the COVID-19 Course 

To analyze SARS-CoV-2 positive rates along the time course of COVID-19, we divided the 4,275 specimens into six disease stage groups, including <0 (102, 2.4%), 0–7 (673, 15.7%), 8–14 (1065, 24.9%), 15–21 (1162, 27.2%), 22–28 (775, 18.1%), >28 (498, 11.6%) d.a.o groups based on the different days between symptom onset and specimen collection (**Table 2**). The SARS-CoV-2 positive rate before symptom onset was 76.5% (**Table 4**). While after symptom onset, the viral positive rate continually declined from 70.1% (472/673) in the first week, to 33.8% (360/1065) in the second week and 24.7% (287/1162) in the third week, and subsequently increased to 31.4 and 33.1% in the fourth week and after (**Table 4**). Taking the positive rate in the first week as a reference, the weekly changes in the positive rates among six stages were statistically significant (**Table 5**). In addition, compared to URT specimens at corresponding stages, sputum showed significantly increased SARS-CoV-2 positive rates in the first week (82.9%) [OR = 2.88, 95%CI (1.18–7.02), *P* = 0.020] and the fourth week (37.7%) [OR = 2.29, 95%CI (1.34–3.92), *P* = 0.003] , but feces/rectal swabs had a significantly lower positive rate later than 28 d.a.o (27.3%) [OR = 0.42, 95%CI (0.21–0.84), *P* = 0.013] (**Table 5**).

**Figure 1:**
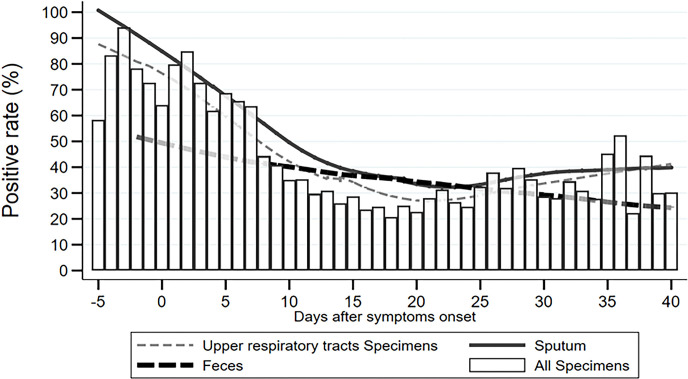
Distribution of SARS-CoV-2 NAT daily positive rates across disease course.

To describe the trend of the SARS-CoV-2 positive rate varying across the time course of COVID-19 more specifically, we calculated the daily positive rates of URT specimens, sputum, feces/rectal swabs, and overall specimens between 5 days before and 40 days after symptom onset. As shown in **Figure 1**, the daily positive rates of overall specimens presented as a flat “U” shape through the disease course, with the highest rates of 85–95% during −3 and 3 d.a.o, the lowest rate of 20% at around 18 d.a.o (**Figure 1**). The second peak was a 52% positive rate at the late stage of 36 d.a.o (**Figure 1**). Both URT specimens and sputum had a similar varying trend of positive rate as overall specimens. The daily positive rates of sputum were higher than those of URT specimens across the entire disease course, except for an overlap later than 36 d.a.o. However, the daily positive rates of feces/rectal swabs continually decreased from about 50% at 2 days before onset, to 25% at 40 days after onset (**Figure 1**).

**Figure 2:**
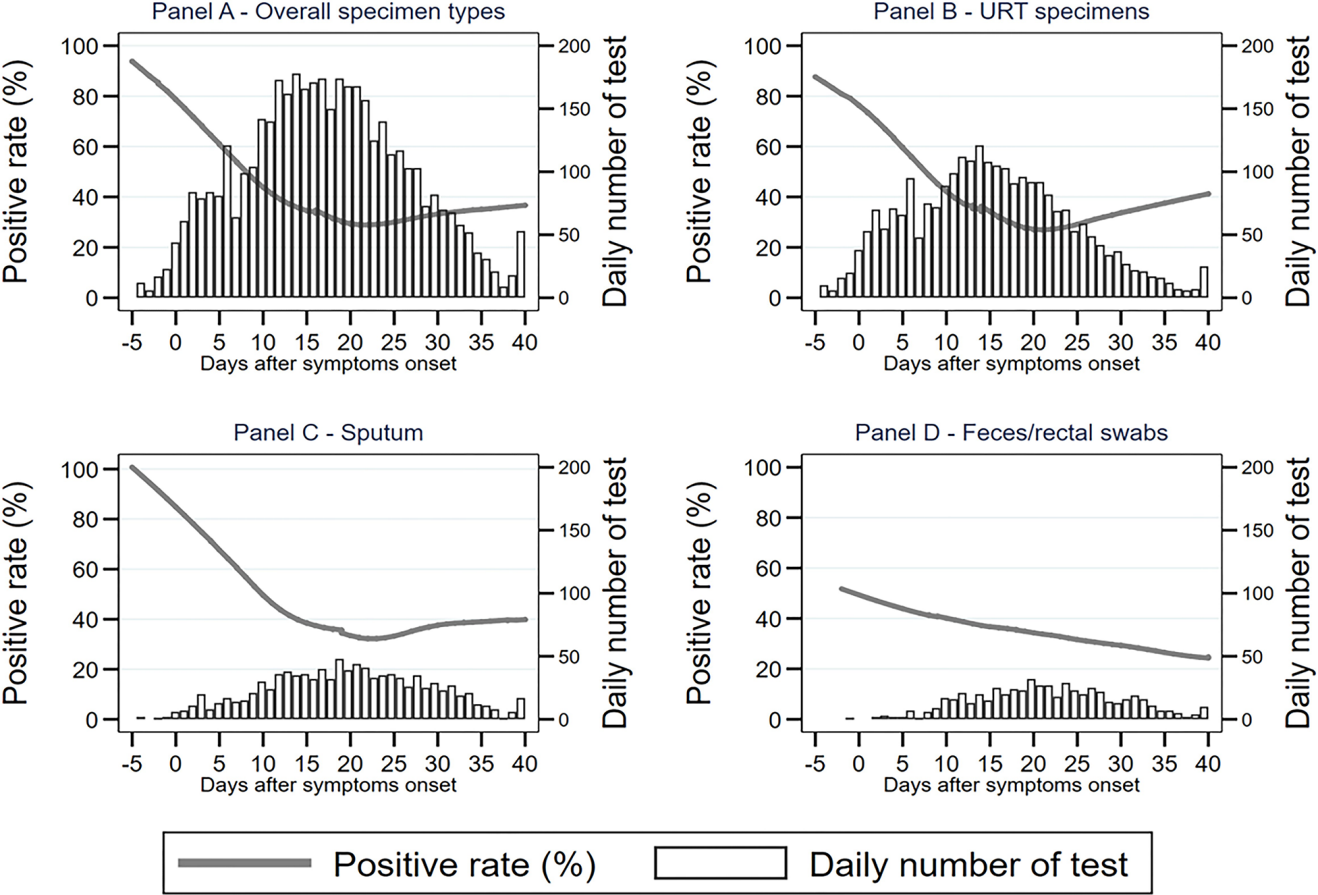
The distribution of positive rates in different specimens across the disease course.

By investigating the number of daily tested specimens between −5 and 40 d.a.o in our study cohort, we revealed there was a normal distribution in overall specimens, as well as subtype groups, URT specimens, sputum, and feces/rectal swabs, across the entire disease course (**Figure 2**), excluding an inference of different specimen distributions.

### Cumulative Positive Rates of SARS-CoV-2 NAT With Consecutive Specimens

The median d.a.o of the first specimen collected from the 532 patients was five with an interquartile range of one d.a.o to 10 d.a.o (**Table 6**). The median d.a.o of the second and third specimens sequentially collected were 13.5 and 15, respectively (**Table 6**). Thus, to find an optimal number of specimens for initial diagnosis of COVID-19 as sampling usually across early and middle stages during practically applying SARS-CoV-2 NAT in clinic, we investigated cumulative positive rates of consecutively collected URT specimens and sputum, which were predominant specimens in our study and also the most common used in clinic.

**Table 6:** Cumulative SARS-CoV-2 NAT positive rates of consecutive specimens and time points of sampling.

No. of consecutive specimens	Total	Throat swabs		*^a^*URT specimen		Throat swabs and sputum		URT*^a^* specimens and Sputum	
	^b^ d.a.o of sampling, median (c IQR)	Cumulative positive rate, %(n/N)	*^b^* d.a.o of sampling, median (*^c^*IQR)	Cumulative positive rate, %(n/N)	*^b^* d.a.o of sampling, median (*^c^*IQR)	Cumulative positive rate, %(n/N)	*^b^* d.a.o of sampling, median (*^c^*IQR)	Cumulative positive rate, %(n/N)	*^b^* d.a.o of sampling, median (*^c^*IQR)
**one specimen**	5 (1,10)	72.1 (338/469)	7 (2,13)	75.0 (381/508)	5 (1,12)	80.4 (419/521)	6 (2,12)	82.7 (440/532)	5 (1,10)
**two specimens**	13.5 (8, 20)	77.6 (364/469)	14 (8, 20)	81.7 (415/508)	14 (8, 19)	86.4 (450/521)	14 (8, 20)	89.7 (477/532)	14 (7, 20)
**three specimens**	15 (11, 21)	80.6 (378/469)	16 (10, 21)	85.2 (433/508)	16 (10, 21)	89.6 (467/521)	16 (10, 21)	93.2 (496/532)	15 (10, 21)

^a^URT, Upper Respiratory Tract.

^b^d.a.o days after onset.

c IQR, interquartile range.

**Figure 3:**
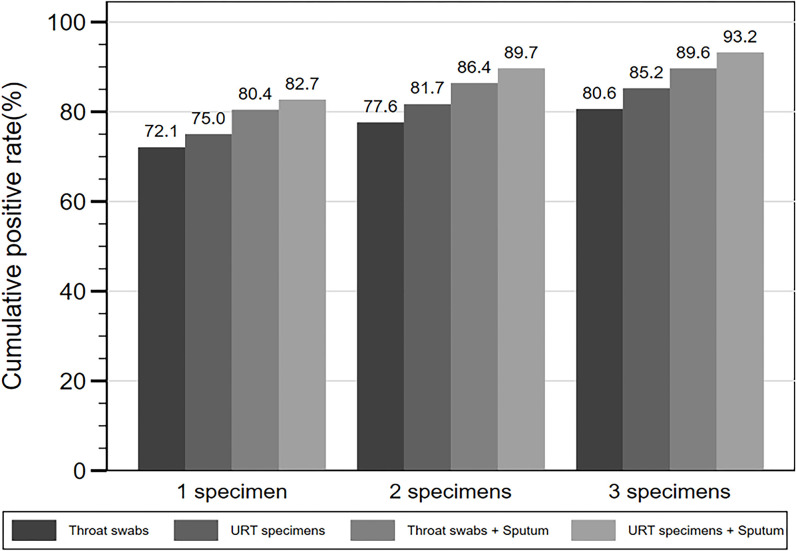
Cumulative SARS-CoV-2 NAT positive rates of consecutive specimens.

Among the 469 patients with throat swabs collected for SARS-CoV-2 NAT, 338 (72.1%) patients were detected positive after testing one swab, 364 (77.6%) after testing two consecutive swabs, and 378 (80.6%) after testing three consecutive swabs (**Figure 3**, **Table 6**). There were 508 patients with URT specimens collected for SARS-CoV-2 NAT. From those, 75.0, 81.7, and 85.2% were detected positive after one, two, and three consecutive specimens, respectively. While for 521 patients tested with either throat swabs or sputum specimens for SARS-CoV-2, the detection rate was 80.4% after one specimen tested, and increased to 86.4 and 89.6% after testing two and three specimens. The detection rate further increased to 82.7 and 89.7% after testing one and two specimens, respectively, for 532 patients with either URT specimens or sputum collection, and eventually reached 93.2% after testing three consecutive specimens (**Figure 3**, **Table 6**). The median d.a.o of the first, second, and third specimens collected in four specimen groups above were 5–7, 14, and 15–16, respectively. There was no significant difference observed among the four specimen groups above, thus excluding a probability of the difference in detection efficiency originated from sampling at different stages (**Table 6**).

## Discussion 

The severity profile of COVID-19 cases included in our study is consistent with that across China reported by the WHO-China joint mission (WHO, 2020). The top three major specimens in the study were throat swabs, sputum, and feces/anal swabs, representing the most common collected specimen types and three different anatomic sites of SARS-CoV-2 viral load.

The overall SARS-CoV-2 positive rate of the 4,275 specimens was 37.5%, which is consistent with recent studies reporting positive rates ranging from 30 to 50% (Wang W. et al., 2020; Wang Y. et al., 2020; Xie et al., 2020). Among the seven specimen types, BALF had the highest positive rate, followed by nasopharyngeal swabs, sputum, throat swabs, nasal swabs, and feces/rectal swabs. The positive rates of three URT specimens, including throat, nasal, and nasopharyngeal swabs showed no significant difference, but were significantly lower than that of sputum, and higher than that of feces/rectal swabs. It suggests that the lower respiratory tracts have higher viral load compared to the upper respiratory tracts. LRT specimens, especially BALF had the highest detection rate for SARS-CoV-2 among all types of specimens, which is consistent with a recent study (Wang W. et al., 2020). However, considering the high infection risk and the low patient acceptance of the BALF collection procedure, it usually applies to critical cases only. Instead, sputum is an alternative LRT specimen type that is the best choice of SARS-CoV-2 NAT for COVID-19 patients with a productive cough. Otherwise, three URT swabs, including throat, nasal, and nasopharyngeal swabs are recommended for SARS-CoV-2 NAT. As the positive rate of feces/rectal swabs, especially blood specimens were significantly lower than that of respiratory specimens, they are not recommended for initial diagnosis of COVID-19.

The positive rate of SARS-CoV-2 NAT was not significantly different among patients with different COVID-19 disease severities, except for critical cases which had a significantly greater positive rate. However, the positive rate of SARS-CoV-2 NAT was significantly different among patients under the improved, stable and aggravated statuses, demonstrating that higher detection rates correspond to disease status. It suggests that SARS-CoV-2 viral load increases with disease status progression. For patients with moderate disease or under the improved status, the positive rate of sputum specimens was significantly higher than that of other specimen types. In contrast, the positive rates of feces/rectal swabs were significantly lower when patients had moderate and critical disease, or under improved status. It suggests that sputum, but not feces/rectal swabs has higher detection efficiency for SARS-CoV-2 NAT than URT specimens for patients in most of disease severities and at most of disease statuses.

Most importantly, our study revealed 85–95% positive rates at 3 days before and after onset of symptoms, but a 20% positive rate at 18 d.a.o. Statistical analysis revealed continually decreasing positive rates during the disease course of −3 to 24 d.a.o. It suggests that viral load peaks at very early stage of the disease, even a few days before symptom onset, but decreases rapidly one week after symptom onset (Pan et al., 2020; Young et al., 2020; Wolfel et al., 2020; Zou et al., 2020). Our results demonstrated that significantly reduced positive rates during the middle and late stages of the disease skew the overall detection rate of SARS-CoV-2 NAT to just 30–40%. However, when sampling as close to symptom onset as possible, the detection efficiency of SARS-CoV-2 NAT reached above 90%, which is high enough for initial diagnosis of COVID-19. Thus, the interval between sampling and symptom onset is the most important factor impacting detection efficiency of SARS-CoV-2 NAT compared to specimen type, disease severity and status. In addition, the second peak in the 5^th^ week of the disease course probably represents a reversion of viral shedding during disease recovery, and could explain the reappearance of positive results in very few patients after two consecutive negative specimens. Interestingly, the positive rate of feces/rectal swabs continually decreased through the entire disease course, suggesting that it might correspond with disease progression as time.

Notably, our results show that testing a single throat swab generated only a 72.1% detection rate for COVID-19 patients. In contrast, three consecutive respiratory specimens, especially after including sputum, could significantly increase the detection rate to 93.2%. It suggests that consecutive respiratory specimens tested could effectively improve the detection efficiency of SARS-CoV-2 NAT, especially if missing the most optimal timepoint to collect specimens.

Some limitations should be known in the current study. First, our results were based on kits from at least five companies; we could not compare the difference between different kits, because we did have these data. Second, process of acquired sample would have an influence on the positive rate; most samples might have been got by nurses or physicians; we could not compare the difference between junior nurse/physicians or senior nurses/physicians. Third, the time span from sampling to testing might be an issue for the study.

Taken together, the study results revealed that the detection efficiency of SARS-CoV-2 NAT varies with specimen type, number of specimens, patient disease severity and status, and especially disease stages of specimen collection. Thus, to improve the detection efficiency of SARS-CoV-2 NAT, we highly recommend: 1) sampling as close to symptom onset as possible for initial diagnosis of COVID-19; 2) consecutively sampling 2–3 respiratory specimens with at least one LRT specimen if missing early stages of COVID-19.

## Data Availability Statement

The raw data supporting the conclusions of this article will be made available by the authors, without undue reservation.

## Author Contributions

LJ, LW, and ZY designed the study. JieZ, KL, and LZ drafted the manuscript and analyzed the data. JianZ, ZR, TS, and HY helped to check the manuscript. All authors contributed to the article and approved the submitted version.

## Funding

This work was supported by the grants from Sichuan Science and Technology Program (2020YFS0014 to ZY) and was supported by the National Natural Science Foundation of China [81670893, 81702064].

## Conflict of Interest

The authors declare that the research was conducted in the absence of any commercial or financial relationships that could be construed as a potential conflict of interest.
